# Effects of the ABC pathway on clinical outcomes in a secondary prevention population of Chinese patients with atrial fibrillation: A report from the Optimal Thromboprophylaxis in Elderly Chinese Patients with Atrial Fibrillation (ChiOTEAF) registry

**DOI:** 10.1002/joa3.12862

**Published:** 2023-05-03

**Authors:** Ameenathul M. Fawzy, Agnieszka Kotalczyk, Yutao Guo, Yutang Wang, Gregory Y. H. Lip, the ChiOTEAF Registry Investigators

**Affiliations:** ^1^ Liverpool Centre for Cardiovascular Science University of Liverpool Liverpool John Moores University and Liverpool Heart & Chest Hospital Liverpool UK; ^2^ Department of Cardiology, Congenital Heart Diseases and Electrotherapy Silesian Centre for Heart Diseases, Medical University of Silesia Zabrze Poland; ^3^ Department of Pulmonary Vessel and Thrombotic Disease Sixth Medical Centre, Chinese PLA General Hospital Beijing China; ^4^ Department of Cardiology Second Medical Centre, Chinese PLA General Hospital Beijing China; ^5^ Danish Center for Clinical Health Services Research, Department of Clinical Medicine Aalborg University Aalborg Denmark

**Keywords:** ABC pathway, atrial fibrillation, prognosis, secondary prevention

## Abstract

**Background:**

The atrial fibrillation better care (ABC) pathway is a simple, comprehensive framework that facilitates provision of integrated care for atrial fibrillation (AF) patients.

**Objective:**

We evaluated management of AF patients in a secondary prevention cohort using the ABC pathway and examined the impact of ABC adherence on clinical outcomes.

**Methods:**

The Chinese Patients with Atrial Fibrillation registry is a prospective registry conducted in 44 sites across China between October 2014 and December 2018. The primary outcome was the composite of all‐cause mortality/any thromboembolism (TE), all‐cause death, any TE and major bleeding at 1 year.

**Results:**

Of the 6420 patients, 1588 (24.7%) had a prior stroke or transient ischemic attack and were identified as the secondary prevention cohort. After excluding 793 patients due to insufficient data, 358 (22.5%) were ABC compliant and 437 (27.5%) ABC noncompliant. ABC adherence was associated with a significantly lower risk of the composite outcome of all‐cause death/TE, odds ratio (OR) 0.28 (95% confidence interval [CI]: 0.11–0.71) and all‐cause death, OR 0.29 (95% CI: 0.09–0.90). Significant differences were not observed for TE, OR 0.27 (95% CI: 0.06–1.27) and major bleeding, OR 2.09 (95% CI: 0.55–7.97). Age and prior major bleeding were significant predictors of ABC noncompliance. Health‐related quality of life (QOL) was higher in the ABC compliant group versus the noncompliant group (EQ score 0.83 ± 0.17 vs. 0.78 ± 0.20; *p* = .004).

**Conclusion:**

ABC pathway adherence in secondary prevention AF patients was associated with a significantly lower risk of the composite outcome of all‐cause death/TE and all‐cause death, as well as better health‐related QOL.

## Atrial fibrillation better care pathway adherence in secondary prevention atrial fibrillation patients was associated with a significantly lower risk of the composite outcome of all‐cause death/thromboembolism and all‐cause death, as well as better health‐related quality of life.

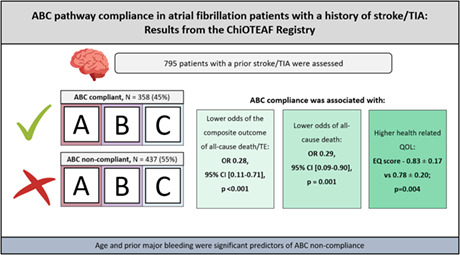

## 1 INTRODUCTION

Atrial fibrillation (AF) and ischemic stroke share an intricate relationship, both risk factors for strokes and each worsening outlook in the presence of the other. AF confers a fivefold increase in the risk of ischemic stroke, while a history of stroke or transient ischemic attack (TIA), alongside AF, is associated with 2.5‐fold risk of a subsequent stroke.^1^, ^2^ Strokes associated with AF carry a poorer prognosis owing to the high degree of functional impairment, permanent disability, and mortality rate.^3^ Hence, primary prevention of stroke is a keystone of AF management.

Given the high recurrence of stroke in AF and the likelihood of a debilitating cerebrovascular event that risks further deterioration, secondary prevention is as crucial and should be a vital element of AF management. However, this carries less emphasis in the literature, with studies specifically focusing on AF patients with previous TIAs/strokes being limited, and little indication of the long‐term clinical outcomes. In a small study by Hayden et al. only 39.2% of AF patients who had ischemic strokes were still alive after 5 years, with a quarter of patients requiring nursing homes and 21.5% having a 5‐year stroke recurrence rate, indicating an extremely poor prognosis and the need for effective measures to ameliorate this.^4^ While this need for a multi‐dimensional approach is realized, existing data suggest suboptimal clinical practice.^5^

Recently, the atrial fibrillation better care (ABC) pathway was introduced as a simple yet comprehensive clinical tool to facilitate provision of integrated care for AF patients, addressing their risk of stroke, symptoms, and cardiovascular risk factors.^6^ The ABC pathway is now recommended in guidelines, having been validated in patient populations from different geographical regions and in specific disease cohorts, all consistently demonstrating favorable clinical outcomes in those who are adherent.^7^, ^8^ Thus, we sought to evaluate management of a secondary prevention cohort (i.e., AF patients with a previous history of stroke or TIA) according to the ABC pathway and examine the impact of ABC adherence on clinical outcomes in an elderly Chinese population.

## 2 METHODS

The protocol of the Optimal Thromboprophylaxis in Elderly Chinese Patients with Atrial Fibrillation (ChiOTEAF) registry has been previously described.^9^ Concisely, the study was conducted between October 2014 and December 2018 in 44 sites from 20 Chinese provinces, enrolling consecutive AF patients (with a documented AF episode within 12 months prior to enrolment) presenting to cardiologists, neurologists, or surgeons. Written informed consent was obtained from all participants.

Follow‐up visits were performed at 6 and 12 months and then annually for the next 2 years. Data were collected by local investigators at enrolment and follow‐up visits (including patient visits and/or chart reviews and/or telephone follow‐up) and recorded in an electronic database.

The registry was approved by the Central Medical Ethics Committee of Chinese PLA General Hospital, Beijing, China (approval no. S2014‐065‐01) and local institutional review boards.

### 2.1 Definitions

The ABC pathway was retrospectively evaluated according to its original definition: patients were considered to have fulfilled the “A” criterion if properly treated with oral anticoagulants (OACs) according to their thromboembolic (TE) risk; “B” criterion if symptoms were adequately controlled, defined by an European Heart Rhythm Association (EHRA) score of I or II (no symptoms or mild symptoms) at the baseline visit; and “C” criterion if they had disease‐specific treatment(s) of any cardiovascular risk factors at baseline. We considered management as guideline‐adherent if the following were true: (i) angiotensin‐converting enzyme (ACE) inhibitors/angiotensin receptor blockers (ARB), calcium channel inhibitors, diuretics, beta‐blockers for hypertension; (ii) ACE inhibitors/ARB, beta‐blockers, and statins for coronary artery disease; (iii) statins for peripheral artery disease; (iv) statins for previous ischemic stroke; (v) ACE inhibitors/ARB and beta‐blockers for heart failure; (vi) insulin or oral antidiabetics for diabetes mellitus; and (vii) statins for lipid disorders. Patients were considered *ABC adherent* when all three criteria (A+B+C) were achieved.^10^ Secondary prevention referred to the management of AF patients with a prior ischemic stroke/TIA.

Other variables and their definitions matched the EURObservational Research Programme Atrial Fibrillation Long‐term General Registry.^11^ CHA_2_DS_2_‐VASc and HAS‐BLED scores were used to assess TE and bleeding risks respectively. Bleeding (intracranial and extracranial) events were categorized based on the International Society on Thrombosis and Haemostasis definition.^12^ The EuroQol five dimensions questionnaire (EQ‐5D‐5L)^13^ was used to assess patient‐reported quality of life (QOL).

### 2.2 Objectives

The primary objective of this analysis was to evaluate the impact of ABC pathway on clinical outcomes at 1 year in a secondary prevention cohort of AF patients with data available to assess ABC management. Endpoints of interest were the composite outcome of all‐cause death/any TE event (including ischemic stroke, TIA, or peripheral embolism), all‐cause death, TE events, and major bleeding.

Secondary objectives were to (i) evaluate the impact of the ABC pathway on clinical outcomes compared with the overall secondary prevention cohort and (ii) identify potential predictors of ABC‐adherent management.

### 2.3 Statistical analysis

Continuous variables were reported as mean ± standard deviation; between‐group comparisons were made using the Student's *t*‐test or the Mann–Whitney *U*‐test (based on distribution). Categorical variables were expressed as counts and percentages; between‐group comparisons were made by chi‐squared test or Fisher's exact test. QOL was assessed using the EQ summary index (range 0–1; score of 1 indicating the best health state), estimated from the EQ‐5D‐5L value set for China.^14^

Logistic regression analysis evaluated the association between the degree of ABC compliance and clinical outcomes, adjusted for clinically significant variables (age, sex, heart failure, chronic kidney disease, chronic obstructive pulmonary disease, first diagnosed AF). For the secondary objective, sensitivity analysis for the overall secondary prevention cohort was performed. Finally, a logistic univariate regression analysis was used to assess the predictors of the ABC compliance; all the significant variables were included in a multivariate regression model. Results were expressed as odds ratios (ORs), 95% confidence intervals (CIs), and *p*‐values. *p* < .05 was considered statistically significant. Statistical analysis was performed using SPSS® version 24 (IBM Corp.).

## 3 RESULTS

**FIGURE 1:**
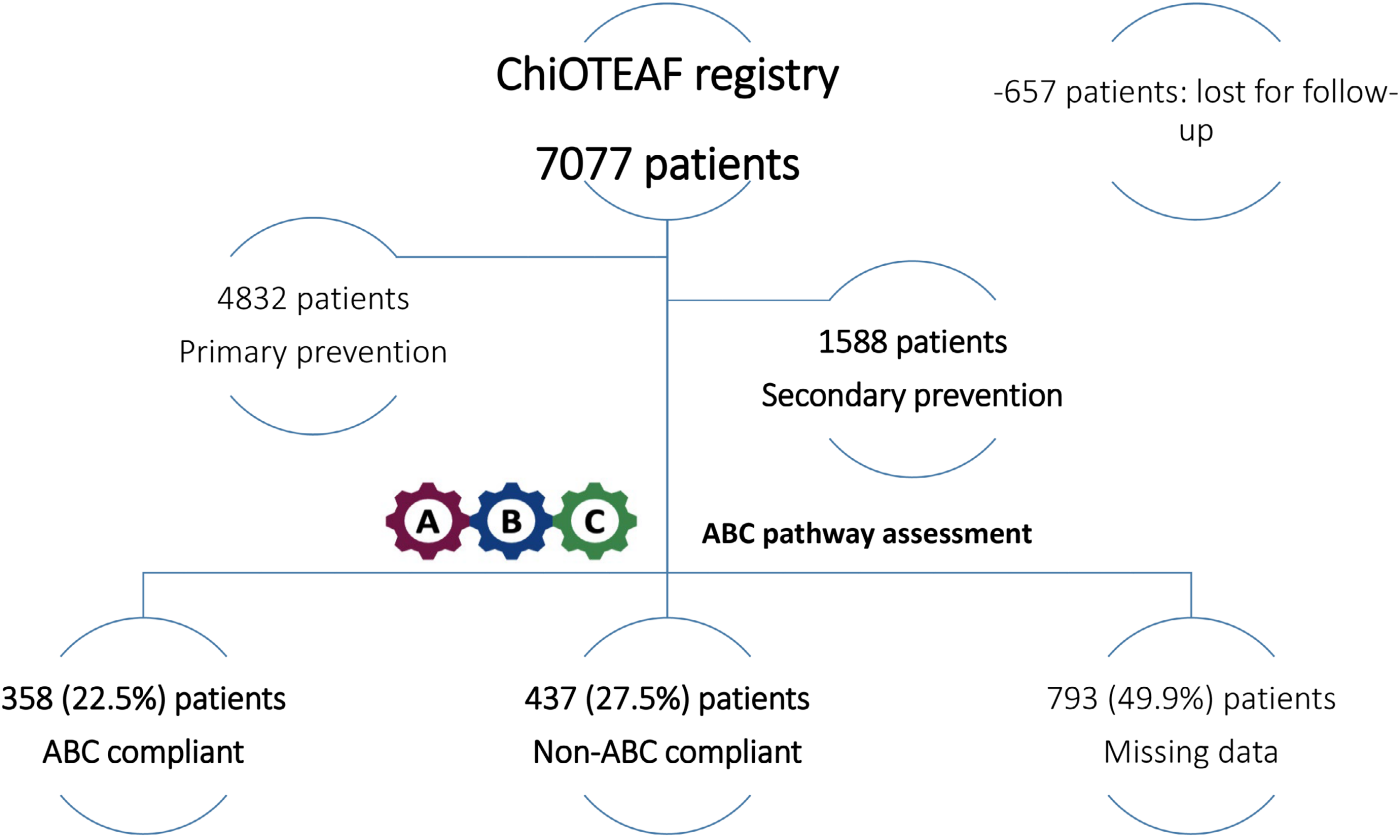
Patient selection process for study.

The ChiOTEAF registry enrolled 7077 patients, of which 657 (9.3%) were lost to follow‐up at 1 year (Figure 1). Only 795 (50.1%) of the 1588 patients with prior ischemic stroke/TIA (mean age 78.6 ± 9.8 years, 36.3% female) had granular data sufficient to assess ABC pathway adherence. Of these, 358 (45.0%) were managed according to the ABC pathway (ABC compliant group) and 437 (55.0%) were not (ABC non‐compliant group). The overall cohort had a high risk of bleeding (mean HAS‐BLED score of 3.0 ± 1.0) and ischemic stroke (mean CHA_2_DS_2_VASc score of 5.3 ± 1.5). Baseline characteristics are reported in Table 1. Patients in the ABC compliant group were younger (73.6 ± 9.8 years vs. 77.8 ± 9.6 years; *p* < .001), with a lower prevalence of chronic kidney disease (10.1% vs. 15.8%; *p* = .018), chronic obstructive pulmonary disease (7.8% vs. 13.5%; *p* = .011), dementia (2.5% vs. 5.5%; *p* = .036), and prior major bleeding (1.7% vs. 8.7%; *p* < .001) compared with the ABC noncompliant patients.

**TABLE 1:** Baseline characteristics of the study cohort.

	Overall (*N* = 1588), *n* (%)	Atrial fibrillation better care (ABC) compliant (*N* = 358), *n* (%)	ABC non‐compliant (*N* = 437), *n* (%)	*p*‐Value
Age^a^ (years)	78.6 ± 9.8	73.6 ± 9.8	77.8 ± 9.6	<.001
Female gender	576 (36.3)	144 (40.2)	157 (35.9)	.214
First diagnosed AF (*n* = 1303)	214 (13.5)	42 (12.0)	71 (16.7)	.065
Paroxysmal AF (*n* = 1303)	504 (31.7)	162 (46.3)	196 (46.1)	.963
Body mass index, kg/m^2^ (*n* = 1587)	24.0 ± 3.6	24.4 ± 3.8	24.2 ± 3.4	.269
Medical history				
Diabetes mellitus (*n* = 1587)	518 (32.6)	106 (29.6)	143 (32.7)	.346
Hypertension (*n* = 1587)	1166 (73.4)	270 (75.4)	307 (70.3)	.104
Heart failure (*n* = 1587)	724 (45.6)	141 (39.4)	199 (45.5)	.081
Coronary artery disease (*n* = 1587)	955 (60.1)	209 (58.4)	279 (63.8)	.115
Chronic kidney disease	267 (16.8)	36 (10.1)	69 (15.8)	.018
Liver disease	65 (4.1)	10 (2.8)	20 (4.6)	.189
Chronic obstructive pulmonary disease	230 (14.5)	28 (7.8)	59 (13.5)	.011
Sleep apnea	79 (5)	21 (5.9)	22 (5.0)	.606
Dementia	141 (8.9)	9 (2.5)	24 (5.5)	.036
Prior major bleeding	122 (7.7)	6 (1.7)	38 (8.7)	<.001
CHA_2_DS_2_VASc^a^ (*n* = 1480)	5.3 ± 1.5	5.0 ± 1.4	5.2 ± 1.5	.074
HAS‐BLED^a^ (*n* = 1481)	3.0 ± 1.0	2.7 ± 1.0	3.1 ± 1.0	<.001
Smoking status (*n* = 1579)				
Former smoker	348 (21.9)	73 (20.4)	88 (20.2)	.723
Active smoker	108 (6.8)	36 (10.1)	37 (8.5)	.439
AF management				
Oral anticoagulation	728 (45.8)	358 (100.0)	60 (13.7)	<.001
Vitamin K antagonists (VKAs)	319 (24.6)	182 (50.8)	30 (6.9)	<.001
Non VKAs	339 (21.3)	176 (49.2)	30 (6.9)	<.001
Antiplatelet	734 (46.2)	81 (22.6)	276 (63.2)	<.001
Amiodarone (*n* = 1585)	162 (10.2)	69 (19.3)	43 (9.8)	<.001
Propafenone (*n* = 1585)	44 (2.8)	16 (4.5)	15 (3.4)	.452
Digoxin (*n* = 1586)	208 (13.1)	36 (10.1)	48 (11.0)	.672
β‐blockers (*n* = 1585)	891 (56.1)	229 (64.0)	245 (56.1)	.024
AF ablation (*n* = 1587)	92 (5.8)	56 (15.6)	19 (4.4)	<.001
Cardiac device (*n* = 1587)	164 (10.3)	32 (8.9)	53 (12.2)	.145
Quality of life				
EHRA score^a^ (*n* = 840)	1.63 ± 0.55	1.62 ± 0.49	1.65 ± 0.59	.403
EQ index^a^ (*n* = 1255)	0.77 ± 0.20	0.83 ± 0.17	0.78 ± 0.20	.004

Abbreviations: AF, atrial fibrillation; CHA_2_DS_2_VASc, congestive heart failure, hypertension, age ≥ 75 [doubled], diabetes, stroke [doubled], vascular disease, age 65–74, female sex; EHRA, European Heart Rhythm Association symptom classification; EQ, health‐related quality of life assessed by the EuroQol five dimensions questionnaire; HAS‐BLED, hypertension, abnormal renal/ liver function, stroke, bleeding history or predisposition, labile international normalized ratio (INR), elderly, drugs/alcohol use.

^a^ Mean ± standard deviation.

### 3.1 AF management and quality of life

All patients in the ABC group were treated with an OAC as opposed to the ABC noncompliant group where only 13.7% were treated with an OAC and 63.2% were treated with an antiplatelet. In the ABC group, a higher proportion of patients had prior AF ablation (15.6% vs. 4.4%; *p* < .001). Health‐related QOL was also higher in this group compared with the noncompliant group (EQ score 0.83 ± 0.17 vs. 0.78 ± 0.20; *p* = .004), but no significant differences in EHRA scores were found.

### 3.2 Clinical outcomes

Compared to the ABC noncompliant group, those in the ABC compliant group had a lower incidence of the composite outcome (1.7% vs. 7.6%; *p* < .001) and all‐cause death (1.1% vs. 5.5% *p* = .001). Odds of the composite outcome (OR: 0.28; 95% CI: 0.11–0.71) and all‐cause death (OR: 0.29; 95% CI: 0.09–0.90) were significantly lower in ABC‐managed patients. No statistically significant differences were observed in the incidence of TE and major bleeding events (Table 2).

**TABLE 2:** The effects of atrial fibrillation better care (ABC) compliance on clinical outcomes.

Outcomes	ABC compliant (*N* = 358), *n* (%)	Non‐ABC compliant (*N* = 437), *n* (%)	*p*‐Value^a^	Odds ratio^c^ (95% CI)
Composite outcome^b^	6 (1.7)	33 (7.6)	<.001	0.28 (0.11–0.71)
All‐cause death	4 (1.1)	24 (5.5)	.001	0.29 (0.09–0.90)
TE events (*n* = 790)	2 (0.6)	10 (2.3)	.075	0.27 (0.06–1.27)
Major bleeding (*n* = 790)	5 (1.4)	5 (1.2)	.765	2.09 (0.55–7.97)

Abbreviations: CI, confidence interval; TE, thromboembolism.

^a^ Between‐group comparison made by chi‐squared or Fisher test.

^b^ Composite outcome of all‐cause death/any thromboembolism.

^c^ Adjusted for age, sex, heart failure, chronic kidney disease, chronic obstructive pulmonary disease, first diagnosed AF.

### 3.3 Exploratory analysis

When we compared baseline characteristics between included (*n* = 795; ABC cohort) and excluded (*n* = 793) patients (Table S1), we found that patients in the ABC cohort were younger (75.9 ± 9.9 years vs. 81.3 ± 8.8 years; *p* < .001) with a lower prevalence of comorbidities, for example, heart failure (42.8% vs. 48.5%; *p* = .022), chronic kidney disease (13.2% vs. 20.4%; *p* < .001), chronic obstructive pulmonary disease (10.9% vs. 18.0%; *p* < .001), dementia (4.2% vs. 13.6%; *p* < .001), and had a higher uptake of OACs (52.5% vs. 39.1%; *p* < .001) and AF ablation (9.4% vs. 2.1%; *p* < .001) compared with the excluded patients.

A lower incidence of the composite outcome (4.9% vs. 21.9%; *p* < .001), all‐cause death (3.5% vs. 19.0%; *p* < .001), TE (1.5% vs. 4.0%; *p* = .003), and major bleeding (1.3% vs. 1.6%; *p* = .006) was observed in the ABC cohort during the 1‐year follow‐up.

### 3.4 Predictors of ABC pathway compliance

On multivariate analysis, (i) prior AF ablation (OR: 3.04; 95% CI: 1.74–5.31) was strongly associated with ABC pathway compliance, whereas (ii) older age (OR: 0.97; 95% CI: 0.96–0.99) and prior major bleeding (OR: 0.24; 95% CI: 0.10–0.60) were independently associated with lower odds of ABC compliance (Table S2).

## 4 DISCUSSION

In this study which evaluated the impact of the ABC pathway on a secondary prevention cohort of AF patients, we found that ABC adherence was associated with a (i) 72% reduction in the odds of the composite outcome encompassing all‐cause death and any TE, (ii) significant reduction in all‐cause death (OR: 0.29; 95% CI: 0.09–0.90), and (iii) higher health‐related QOL compared with ABC nonadherence. These findings advocate the adoption of an integrated care approach such as the ABC pathway for management of this high‐risk group.

To our knowledge, this is the first study to evaluate the efficacy of the ABC pathway in a secondary prevention AF cohort. Although the ABC pathway has been validated and consistently demonstrated cardiovascular and mortality benefits in several AF populations, it has not specifically been evaluated in AF patients with previous strokes or TIAs.^8^ Holistic management of these patients is often overlooked, despite their poor prognosis.

Our study comprised a population with a mean CHA_2_DS_2_VASc score of 5.3 ± 1.5, yet only 45% were managed using the ABC approach. This is consistent with, if not better than, existing studies that indicate that the ABC pathway is followed in just one in five AF patients.^8^ Those managed according to it were younger, with a lower burden of certain risk factors such as diabetes and had comparatively lower CHA_2_DS_2_VASc and HAS‐BLED scores. Arguably, it is the higher risk patients who stand to achieve the most benefit from appropriate risk reduction measures.

Our analysis demonstrated that ABC noncompliance was strongly influenced by age and prior major bleeding, when existing evidence suggests there is no significant increase in the OAC‐associated recurrent bleeding risk once causative factors have been addressed, and potentially with non‐vitamin K antagonists (VKAs) compared to VKAs.^15^, ^16^ In the meta‐analysis by Proietti et al. though OAC resumption was associated with a higher risk of major bleeding, there was no increase in the recurrence of the index bleeding event and a net clinical benefit was still observed due to reduction in all‐cause mortality and TE.^17^ Patients with previous intracranial hemorrhage have also been shown to have a higher risk of subsequent ischemic strokes, particularly in the 3–6 months following the bleeding event, indicating need for optimal secondary prevention.^18^ Indeed, there may be a small population with a true contraindication to OAC and, in such cases, extra emphasis on the “B” and “C” components is prudent.

In the ABC noncompliant group, 29.9% were not prescribed an OAC and 63.2% were prescribed aspirin. While the latter may be perceived as a “less risky” substitute for anticoagulation, it is possible that the aspirin was prescribed for coronary artery disease which was highly prevalent in this group. In any case, with the ample evidence demonstrating that aspirin confers a similar or higher bleeding risk compared to OACs without the protective effects against strokes, it can be inferred that a significant proportion of these patients would have been able to tolerate anticoagulation.^19^ Unfortunately, it was not possible to examine this in detail or determine if those who were not prescribed an OAC had a true contraindication.

ABC pathway adherence was associated with a significant reduction in the risk of composite outcome of all‐cause death and TE, primarily driven by the reduction in all‐cause death. This is consistent with existing studies that have evaluated performance of the ABC pathway, demonstrating not just a reduction in all‐cause mortality but also other outcomes such as TE, ischemic stroke and major bleeding.^8^ Our study, however, showed no significant differences between the two groups for TE and major bleeding, likely due to the small number of events observed, resulting in wide 95% CIs.

Hence, we conducted an exploratory analysis comparing outcomes between the ABC compliant group and the overall cohort comprising ABC noncompliant patients and those whose ABC compliance status could not be fully evaluated. Outcome events were larger in number, albeit still small, and reflected findings from the primary analysis. In the study by Wang et al. a subgroup analysis of patients with previous strokes or TIAs demonstrated a 55% ABC compliance rate with a nonsignificant reduction in all‐cause death, hazard ratio 0.68 (95% CI: 0.47–1.00), bearing in mind the small cohort size.^20^

ABC compliance has been associated with a reduction in both the TE and bleeding risks.^8^ With every increase in the number of ABC criteria fulfilled, a progressive reduction in the risk of clinical outcomes has been illustrated in existing studies, evidencing that an integrated care approach comprising of symptom management and risk factor control are just as crucial as anti‐coagulation.^20^, ^21^ Our study shows that qualifying for three ABC pathway criteria was independently associated with a reduction in all‐cause death/ any TE event. However, it was not possible to perform a separate analysis according to the number of fulfilled ABC criteria and each criterion, due to the small sample size and the low rate of adverse events. We investigated these in our recent study of the overall ChiOTEAF cohort.^10^ Romiti et al.^22^ specifically evaluated the impact of each criterion in the ABC pathway in the GLORIA‐AF cohort. Adherence to just the “B” criterion was not associated with a significant reduction in the risk of the primary outcome of all‐cause death and major adverse cardiovascular events and adherence to the “A and C” criteria had a significantly lower risk compared to the “B and C” criteria. Nonetheless, the number of adverse events progressively decreased as the number of ABC criteria fulfilled increased with the lowest risk in those that were fully ABC adherent, highlighting that each component in the ABC pathway acts synergistically, and a holistic approach to AF management is best to improve patients' outcomes.^22^, ^23^ Thus, active measures to reinforce its uptake including education and awareness amongst healthcare professionals is necessary to better patient prognosis.

### 4.1 Limitations

Although the ChiOTEAF registry was prospective because it was not specifically designed to examine ABC pathway, our analysis was conducted retrospectively in accordance with the published definitions for each of the components.^6^ Consequently, nearly half of the secondary prevention cohort had to be excluded due to insufficient data for ABC compliance assessment, rendering a potential source of bias. Furthermore, the lack of granularity of data meant it was not possible to explore certain aspects such as the etiology of prior bleeding events, reasons for aspirin prescription and OAC preclusion. Due to the small number of patients in each group and short duration of follow‐up, the number of observed events for clinical outcomes such as major bleeding were small and meaningful inferences cannot be drawn for these. For this reason, we were also unable to evaluate outcomes according to the number of ABC compliant criteria. Lastly, despite half the patients being on VKAs, data on time in therapeutic range which can significantly influence outcomes were unavailable.

## 5 CONCLUSION

ABC pathway adherence in the high‐risk secondary prevention AF cohort was associated with a significantly lower risk of the composite outcome of all‐cause death/TE and all‐cause death, as well as better health‐related QOL.

## AUTHOR CONTRIBUTIONS

This paper has not been submitted for publication to any other journal. All authors have made a significant contribution and have read and approved the final draft. Ameenathul M. Fawzy, Agnieszka Kotalczyk, and Yutao Guo contributed equally to design the study, interpreted the data, and drafted the manuscript (joint first authors). Yutang Wang and Gregory Y.H. Lip contributed in the interpretation of data and revised the manuscript critically for important intellectual content (joint senior authors).

## FUNDING INFORMATION

The study was supported by Beijing Natural Science Foundation, China (Z141100002114050), and Chinese Military Health Care (17BJZ08).

## CONFLICT OF INTEREST STATEMENT

Gregory Y.H. Lip: Consultant and speaker for BMS/Pfizer, Boehringer Ingelheim, and Daiichi‐Sankyo. No fees are received personally. The other authors have no conflict of interest.

## ETHICS STATEMENT

This study was performed in line with the principles of the Declaration of Helsinki. Ethics approval was granted by the Central Medical Ethic Committee of Chinese PLA General Hospital (approval no. S2014‐065‐01).

## CONSENT TO PARTICIPATE

Written informed consent was obtained from all individual participants included in the study.

## Supporting information


Table S1.
Table S2.
ChiOTEAF Registry Investigators

## Data Availability

The datasets used and analyzed during the current study are available from the corresponding author on reasonable request.
